# Serum Therapy in Cancer

**Published:** 1909-10

**Authors:** 


SERUM THERAPY IN CANCER—Erysipelas and Prodigiosus Toxins (Coley).—This product consists of the unfiltered toxins of the streptococcus of erysipelas and the bacillus prodigiosus and is made according to the formula and directions of Dr. Wm. B. Coley, of the New York Cancer Hospital. It is used exclusively for the treatment of inoperable malignant growths, particularly sarcomata, and is administered hypodermatically, in or near the tumor, beginning with one-half to one minim and gradually increasing. Dr. Coley and others report a number of cures; indeed, of spindle-celled sarcomata treated, 50 per cent are said to disappear. Parke, Davis & Co. will send literature free on request.

---

An intractable non-gonorrheal cystitis in the male nearly always indicates a tuberculous kidney.—*American Journal of Surgery.*

